# Feasibility of combining oncology surgery with bariatric surgery; a two-patient case series of sleeve gastrectomy with cytoreductive surgery and HIPEC

**DOI:** 10.1093/jscr/rjab588

**Published:** 2022-01-17

**Authors:** Mohammad Aburahmah, Talal M Hijji, Lama Tareq Saif, Dana Kalagi, Ayman Zaki Azzam, Tarek Amin

**Affiliations:** Surgical Oncology Department, Oncology Center, King Faisal Specialist Hospital and Research Center (KFSH&RC), P. O. Box 3354, Riyadh 11211, Saudi Arabia; College of Medicine, Alfaisal University, P.O. Box 50927, Riyadh 11533, Saudi Arabia; College of Medicine, Alfaisal University, P.O. Box 50927, Riyadh 11533, Saudi Arabia; King Faisal Specialist Hospital and Research Center (KFSH&RC), P. O. Box 3354, Riyadh 11211, Saudi Arabia; Surgical Oncology Department, Oncology Center, King Faisal Specialist Hospital and Research Center (KFSH&RC), P. O. Box 3354, Riyadh 11211, Saudi Arabia; General Surgery Department, Faculty of Medicine, Alexandria University, Alexandria, Egypt; Surgical Oncology Department, Oncology Center, King Faisal Specialist Hospital and Research Center (KFSH&RC), P. O. Box 3354, Riyadh 11211, Saudi Arabia

## Abstract

Patients with an oncologic disease requiring cytoreductive surgery and hyperthermic intraperitoneal chemotherapy may also present with morbid obesity. In some patients, it may be possible to offer bariatric surgery such as sleeve gastrectomy in combination with their cancer resection to treat both diseases concurrently. Two such cases are described where sleeve gastrectomy was done alongside the primary oncologic surgery in the same procedure. Our patients had long-term follow-ups and their overall outcomes were favorable. They achieved remission and acceptable levels of weight loss over their several years of follow-up appointments. The added benefit of bariatric surgery may decrease long-term morbidity and mortality in carefully selected patients. More studies are indicated to fully understand the risks of benefits of this combined procedure in order to offer it on a wider scale.

## INTRODUCTION

Intraperitoneal carcinomatosis is considered among the greatest oncologic challenges, and until recently, regarded to uniformly have a bad prognosis in all cases. As a result of a global effort to discover better treatment options, advancements and refinements in the combined treatment approach of cytoreductive surgery (CRS) and hyperthermic intraperitoneal chemoperfusion (HIPEC) were made [1, 2]. The combination of CRS with HIPEC has now become widely accepted as the standard-of-care treatment method for peritoneal metastases from various oncologic pathologies [3, 4]. However, owing to the morbidity and mortality associated with the procedure, patient selection remains to be an important aspect when considering the use of CRS and HIPEC to ensure a survival benefit without increasing morbidity in patients with extensive disease. [5]

Morbid obesity has been established as a major risk factor in several kinds of malignancies [6]. The possibility of combining the principles of both bariatric surgery and oncologic surgery at the same intervention may exist in carefully selected patients. One way this could be achieved is by combining a potentially curative cancer resection with a sleeve gastrectomy. Thus, attaining the curative outcome of the oncologic procedure along with the benefits of the sleeve gastrectomy in correcting comorbidities and reducing long-term morbidity and mortality [7, 8].

In our paper, we present two such cases in which sleeve gastrectomy was done alongside CRS and HIPEC in a single procedure at our institution. We were able to demonstrate safety and discuss the outcomes, as well as considerations for selection factors in such patients. To the best of our knowledge, this is among the first case series of two patients in which CRS and HIPEC was combined with a bariatric sleeve gastrectomy in a single operation. However, one previous case report has been published [9]. Our cases have the added benefit of long follow-ups periods in patients who have not had previous bariatric procedures.

## CASE PRESENTATION

### Case 1

The patient is a 46-year-old female, referred to our hospital after being diagnosed with a low-grade appendicular mucinous carcinoma with positive mucosin in the peritoneal cavity at another institution. She had initially presented to the primary institution with symptoms of acute appendicitis and underwent an appendectomy where the initial biopsy was taken. She was then referred to our center for further management. The patient was further evaluated at our institution and the decision was taken to undergo CRS with HIPEC. Furthermore, the patient had a medical history significant for morbid obesity with an initial weight of 126.5 kg, height of 1.58 m and body mass index (BMI) of 50.67 kg/m^2^, diabetes mellitus type 2 on an oral hypoglycemic agent, dyslipidemia and hypothyroidism. Upon evaluation, she had met the necessary criteria of the internal consensus conference [10] for the bariatric procedure and after counseling the patient, informed consent was taken to include sleeve gastrectomy along with CRS and HIPEC.

The patient was taken to surgery for CRS and HIPEC by the surgical oncology team. Laparotomy followed by anterior peritonectomy was done. There was diffuse mucinous carcinomatosis particularly on the right diaphragm, the liver surface particularly on the right side, the lesser omentum near the gallbladder, and in the pelvis on the bladder peritoneum. Ovaries were also involved, and there were also multiple tumor deposites on the small bowel and colon.

Sleeve gastrectomy was performed by the bariatric surgery team, removing greater curvature using stapler on Bougie size 36 along the lesser curvature creating the usual tight sleeve used for bariatric surgery. Lesser sac was cleared of all tumor nodules. Peel of the right diaphragm, peritoneum and large parts of the Glisson capsules and removal of the subhepatic peritoneum were done. The surgery also included: Splenectomy, dissection of the peritoneum of the left diaphragm, cholecystectomy, extraperitoneal pelvic dissection, hysterectomy and bilateral salpingo-oophorectomy. HIPEC was finally performed with mitomycin C for 90 minutes. The patient was sent to the intensive care unit (ICU) in a satisfactory condition. The estimated blood loss was 700 cc.

Unfortunately, her hospital course was complicated by wound infection treated by IV antibiotics, and a right internal jugular vein thrombosis with pulmonary embolism which were treated with a full dose of anticoagulation therapy.

Surgical pathology showed negative malignancy in the resected organs with negative margins, and the oncology team recommended no further need for chemotherapy or radiotherapy. The patient was discharged in good condition with regular follow-ups according to protocol: Every 3 months in the first year, every 6 months in the second year, then once yearly. The patient returned to follow-ups 4 years post the operation and has been in remission since the procedure with no complaints. No evidence of leakage, obstruction, hernias or malnutrition were noted postoperatively. In addition to her follow-up appointments with oncology teams she received follow-up from the bariatric team and clinical dietician. Her BMI has steadily decreased from 50.67 kg/m^2^ to 37.6 kg/m^2^ during the first year of follow-up appointments (~68% excess weight loss). However, the patient experienced some weight regain in the following years owing to decreased compliance to lifestyle modifications reaching a final BMI of 42.6 kg/m^2^ on the last follow-up.

### Case 2

The patient is a 32-year-old female with a history of total abdominal hysterectomy and bilateral oophorectomy in 2009 for a primary ovarian tumor, followed by resection of the recurrent tumor with invasion to colon and bladder requiring rectal resection and partial cystectomy in 2015. The patient presented with new highly suspicious lesions on follow-up positron emission tomography-computed tomography (CT) described as avid enlarging focal hyperdense peritoneal soft tissue lesion at the anterior left iliac fossa. The patient also had nodules around the spleen which were thought to be stable. Additionally, the patient was suffering from morbid obesity with a BMI of 47.9 and hypothyroidism. After a thorough explanation to the patient, consent was obtained to undergo CRS with HIPEC, splenectomy, cholecystectomy, appendectomy, distal pancreatectomy, omentectomy, resection of rectosigmoid anastomosis as well as a sleeve gastrectomy. The surgery was performed by our experienced surgical oncology and bariatric surgery teams as in the first case. Blood loss was about 500 cc. She was then admitted to the ICU for post-op management.

The patient’s hospital course was complicated by a major bilateral pulmonary embolism second day postoperatively and developed respiratory failure and was placed on a heparin infusion. She also had a subcapsular hepatic hematoma, portal vein thrombosis, renal infarction and splenectomy bed collection on CT scan. Further management included inferior vena cava filter, wound debridement, abdominal closure with mesh and removal of infected hematoma.

The patient achieved remission initially but unfortunately, developed a recurrence in the colon after 18 months which was complicated by bowel obstruction necessitating a second CRS + HIPEC. The patient had a drastic decrease in BMI initially but is now back in remission with a BMI of 31 kg/m^2^ during her last follow-up (~55% excess weight loss from her initial weight) and is doingwell.

## DISCUSSION

Our series shows that incorporating sleeve gastrectomy to patients undergoing CRS+ HIPEC may be considered a viable option in selective patients to aid in weight reduction post surgery. Provided that the patients are adequately counseled about the risks and benefits, the addition of laparoscopic sleeve gastrectomy could lead to satisfactory results with acceptable added risks.

To the best of our knowledge, our case report is the first case series reporting a combined CRS with HIPEC and sleeve gastrectomy in one intervention with a prolonged follow-up. The previously reported case report suggested a need for the right sequencing of interventional steps wherein the administration of HIPEC was done before the stomach is stapled to reduce the risk of tumor cell entrapment within the gastric staple line [9]. However, the approach used in our series involved performing the sleeve gastrectomy first followed by the use of a HIPEC. The theory suggested in the previous study was that HIPEC should be administered prior to the stapling of the stomach to minimize the possibility of tumor cell entrapment within the gastric staple line. It remains in question whether the sequencing of these interventional steps is necessary to reduce the risk of recurrence.

Patient selection criteria for both sleeve gastrectomy and HIPEC/CRS were met in our patients. Thus, the combined bariatric and surgical oncologic procedure in a single operation was shown to be safe in both of our patients.

Although complex postoperative care was needed; no unpredictable adverse events attributed to the combined procedure occurred in our patients and both were discharged in satisfactory conditions and were compliant with instructions. In addition to the usual complication associated with CRS and HIPEC, complications such as leakage from sleeve gastrectomy should also be monitored. There is a risk of pneumonia and venous thromboembolism for both bariatric and surgical oncologic procedures [11, 12]. Therefore, patients in this setting must receive postoperative management from a team of expert bariatric surgeons and oncologists in addition to clinical dieticians in order to minimize adverse effects and achieve desired outcomes.

The patients presented in the series involved complex cases with a high Peritoneal Cancer index, yet performing the sleeve gastrectomy still proved to be safe and effective. There remains the need for further studies to fully evaluate whether there are improved outcomes for such patients when adding the sleeve gastrectomy in terms of morbidity and mortality. Keeping in mind that sleeve gastrectomy may have an impact on the patient’s nutritional status and ability to heal. We have summarized some key points regards both patients in Table 1. Both patients achieved acceptable weight loss during their follow-up period and a summary of BMI progression is shown in Table 2.

**Table 1:** Summary of cases: patient demographics, operation information and complications

	Case 1	Case 2
Date	11 December 2016	21 October 2018
Age	46	32
Sex	Female	Female
Initial BMI	50.67 kg/m^2^	47.9 kg/m^2^
Comorbidities	Diabetes mellitus type-2 on oral hypoglycemic agents Dyslipidemia Hypothyroidism	Hypothyroidism
ASAc classification	III	III
Tumor type	Mucinous appendicular carcinomatosis	Ovarian tumor with metastatic rectosigmoid cancer and peritoneal metastasis
Tumor stage	Low-grade appendiceal mucinous neoplasm + pseudomyxoma peritonei	IIIC
Surgical technique	HIPEC Cytoreductive surgery Omentectomy Peritonectomy Splenectomy Cholecystectomy Hysterectomy Bilateral salpingo-oophoorectomy Sleeve gastrectomy	HIPEC Splenectomy Appendectomy Distal pancreatectomy Omentectomy Cholecystectomy Reresection of rectosigmoid anastomosis with lymph node dissection Partial gastrectomy
Operative time	8 hours	9 hours 43 minutes
Intraoperative complications	None	None
Complications	Wound infection Right jugular vein thrombosis and gross pulmonary embolism in the main pulmonary artery	Bilateral PE Subcapsular hepatic hematoma and Portal vein thrombosis Renal infarction
Management of complications	IV antibiotics Anticoagulation	Anticoagulation IVC filter Wound debridement, Abdominal closure with mesh Removal of infected hematoma.

**Table 2:** Progression of BMI in both cases over follow-up period

BMI	Case 1	Case 2
Initial BMI	50.67 (12 December 2016)	47.9 (17 October 2018)
6 months	39.6 (19 June 2017)	34.4 (14 April 2019)
12 months	37.7 (16 November 2017)	28.7 (17 September 2019)
24 months	40.2 (12 November 2018)	23.1 (23 October 2020)
36 months	39.5 (27 June 2019)	28 (12 April 2021)
48 months	40.2 (11 November 2019)	–
Last visit	42.6 (12 July 2021)	31.2 (13 June 2021)

## CONCLUSION

Planning a course of treatment for cancer patients with morbid obesity requiring laparotomy presents a major challenge for bariatric surgeons and surgical oncologists. Intraoperative collaboration, proper preoperative evaluation and post-op follow-up are necessary to ensure safe intervention. Patients who are to undergo this combined procedure may be selected based on clinical features such as young age, favorable prognosis, good performance status. Patients need to be adequately counseled regarding both operations, and must be willing to strictly adhere to postoperative instructions.
